# Differences in autonomic nervous system activity between long-acting injectable aripiprazole and oral aripiprazole in schizophrenia

**DOI:** 10.1186/s12888-023-04617-y

**Published:** 2023-03-03

**Authors:** Saki Hattori, Akira Suda, Ikuko Kishida, Masatoshi Miyauchi, Yohko Shiraishi, Nobuhiko Noguchi, Taku Furuno, Takeshi Asami, Mami Fujibayashi, Natsuki Tsujita, Chie Ishii, Norio Ishii, Takashi Saeki, Tadashi Fukushima, Toshio Moritani, Yusuke Saigusa, Akitoyo Hishimoto

**Affiliations:** 1 Department of Psychiatry, Yokohama City University School of Medicine, 3-9 Fukuura, Kanazawa-ku, Yokohama, 236-0004 Kanagawa Japan; 2 Fujisawa Hospital, 383 Kotsuka Fujisawa, Yokohama, 251-8530 Fujisawa Japan; 3 Division of Physical and Health Education, Setsunan University, 17-8 Ikedanakamachi, Neyagawa, 572- 8508 Osaka Japan; 4 Graduate School of Human and Environmental Studies, Kyoto University, Yoshidanihonmatsucho, Sakyo-ku, Kyoto, 606-8316 Japan; 5 Asahinooka Hospital, 128-1 Kawaihonchou, Asahi-ku, Yokohama, 251-8530 Kanagawa Japan; 6 Department of Biostatistics, Yokohama City University School of Medicine, 3-9 Fukuura, Kanazawa-ku, Yokohama, 236-0004 Kanagawa Japan

**Keywords:** autonomic nervous system, schizophrenia, aripiprazole, long-acting injectable aripiprazole

## Abstract

**Background:**

Distinct oral atypical antipsychotics have different effects on autonomic nervous system (ANS) activity. Among them, oral aripiprazole has been linked to dysfunction of the ANS in schizophrenia. Long-acting injectable aripiprazole is a major treatment option for schizophrenia, but the effect of the aripiprazole formulation on ANS activity remains unclear. In this study, we compared ANS activity between oral aripiprazole and aripiprazole once-monthly (AOM) in schizophrenia.

**Methods:**

Of the 122 patients with schizophrenia who participated in this study, 72 received oral aripiprazole and 50 received AOM as monotherapy. We used power spectral analysis of heart rate variability to assess ANS activity.

**Results:**

Patients who received oral aripiprazole showed significantly diminished sympathetic nervous activity compared with those who received AOM. Multiple regression analysis revealed that the aripiprazole formulation significantly influenced sympathetic nervous activity.

**Conclusion:**

Compared with oral aripiprazole, AOM appears to have fewer adverse effects, such as sympathetic nervous dysfunction.

## Background

The mortality risk of patients with schizophrenia exceeds that of the general population by two to three times [1], and cardiovascular disease is a major cause of death [2]. Notably, a general association has been found between sudden cardiac death and diminished autonomic nervous system (ANS) activity as well as overall morbidity [3]. Patients with schizophrenia are known to exhibit lower ANS activity compared with general populations [4, 5] and antipsychotic medications can lead to exacerbation of ANS dysfunction [6–8]. In previous studies on schizophrenia, we reported the dose-dependent manner by which antipsychotic drugs significantly decrease ANS activity [8] as well as the dissimilar effects of different atypical antipsychotics on ANS activity, specifically that the quetiapine group showed significantly diminished sympathetic and parasympathetic activity compared with the risperidone and aripiprazole groups and significantly lower sympathetic activity relative to olanzapine [9].

Long-acting injectable (LAI) antipsychotics are a useful option for relapse prevention, especially when patients with schizophrenia are nonadherent to their prescribed medication [10–14]. A recent study reported that atypical LAI antipsychotics had the lowest risk of all-cause mortality compared with other types of antipsychotics [15]. In addition, patients receiving atypical LAI antipsychotics had the lowest incidence of cardiovascular death among patients with schizophrenia who were receiving typical LAI antipsychotics, oral typical antipsychotic, or oral atypical antipsychotics [15]. However, the mechanism associated with the advantage of LAI antipsychotics over oral antipsychotics in relation to cardiovascular deaths is still unclear. In particular, the effect of each atypical LAI antipsychotic on ANS activity remains to be investigated. Moreover, few studies have elucidated the differences in ANS activity between the LAI and oral formulations of the same antipsychotic.

We focused on aripiprazole because it is a representative drug for treating schizophrenia and has both oral and LAI formulations. Aripiprazole once-monthly (AOM) is a major LAI antipsychotic prescribed for schizophrenia. Aripiprazole, a second-generation antipsychotic with a high-affinity partial agonist of dopamine D2 receptors and serotonin 5-HT1A receptors and an antagonist of 5-HT2A receptors [16], is efficacious in both the short and long term [17], with a low incidence of side effects (e.g., weight gain and hyperprolactinemia) and metabolic adverse events, except for akathisia [18, 19]. However, even though AOM is a major option in the treatment of schizophrenia, its effects on ANS dysfunction compared with those of oral aripiprazole are unclear and warrant further research.

In this study, we investigated the effects of oral aripiprazole and AOM on ANS activity. We noninvasively evaluated ANS activity by assessing 5-min resting heart rate variability (HRV), which can reflect autonomic nervous imbalance, in line with our previously reported work on the effects of oral atypical antipsychotics on ANS activity and their pharmacogenetic effects on ANS activity in schizophrenia [9, 20, 21]. The reliability, validity, and practicability of HRV have been described [22]. Understanding differences in the effects of oral and LAI aripiprazole on ANS activities could help clinicians decide which formulation to use in patients with schizophrenia.

## Methods

### Participants

This study used a cross-sectional design in a consecutive sample of 122 Japanese patients with schizophrenia (10 inpatients, 112 outpatients; 51 men, 71 women; mean age ± standard deviation, 43.2 ± 13.5 years) who were treated at Fujisawa Hospital, Asahinooka Hospital, or Yokohama City University Hospital in Japan from June 2016 to August 2019. All patients had received oral aripiprazole as monotherapy or AOM as monotherapy for more than 3 months at the same dosage and without adjustment in the previous 3 months. Fifty-two patients in the oral aripiprazole group and 9 patients in the AOM group were those involved in our previous study [9, 23]. In addition, patients with schizophrenia receiving oral aripiprazole or AOM were newly recruited for the present study. Exclusion criteria were as follows: nonadherence to medication prescribed (evaluated using medical interviews); current or previous cardiovascular, respiratory, neurological, or endocrine illness; and current or previous substance abuse that could obscure diagnosis. Patients taking non-psychotropic medications such as antihypertensive drugs other than laxatives, which do not affect HRV, were excluded. Psychiatrists with sufficient clinical experience made the diagnosis of schizophrenia according to the criteria of the Diagnostic and Statistical Manual of Mental Disorders, 4th edition [24]. They also evaluated patients’ positive, negative, and general signs using a Japanese translation of the Positive and Negative Syndrome Scale (PANSS; [25]) to determine symptom severity on the same day as an electrocardiogram (ECG) was recorded.

We collected participants’ clinical information from the medical records and calculated the doses of all psychotropic medications prescribed, including aripiprazole, anticholinergic, and benzodiazepine agents, using conversions to standard equivalents of chlorpromazine, biperiden, and diazepam [26].

This study was approved by respective ethics committees of Fujisawa Hospital, Asahinooka Hospital, and Yokohama City University Hospital and was conducted in accordance with the Declaration of Helsinki. Informed consent was obtained from all participants following a full explanation of the study.

### R–R interval power spectral analysis

As described in detail in our previous studies [9, 20, 21, 27], we performed computer-assisted measurement of 5-min resting HRV to noninvasively evaluate ANS activity. Briefly, participants abstained from caffeine consumption and smoking from the time of waking up on the measurement day. All experimental sessions were held between 9 am and noon. After initially resting for at least 10 min, patients were seated for 5 min for the ECG.

In the HRV power spectral analysis, a series of sequential R–R intervals obtained from the 5-min ECG was decomposed via fast Fourier transform into a sum of sinusoidal functions of different amplitudes and frequencies [28]. As in our previous studies [21, 28], spectral power was quantified in the frequency domain by calculating the areas under the curve in the low frequency band (LF; 0.03–0.15 Hz), reflecting both sympathetic and parasympathetic nerve activity; the high-frequency band (HF; 0.15–0.40 Hz), reflecting mainly parasympathetic nerve activity; and the total power band (TP; 0.03–0.40 Hz), reflecting overall ANS activity.

### Statistical analysis

We used Student’s *t*-test to examine differences in clinical characteristics (age, body mass index [BMI], disease duration, aripiprazole dose, anticholinergic agent dose, benzodiazepine agent dose, and PANSS score, and the LF, HF, and TP components of HRV). We used the chi-square test to examine the ratios of males to females, inpatients to outpatients, and smokers to non-smokers. The association of clinical factors with ANS activity was determined using multiple regression analysis, with the LF, HF, and TP components of HRV considered dependent variables, and age, sex, BMI, aripiprazole dose, anticholinergic dose, benzodiazepine dose, and aripiprazole formulation (oral or LAI) considered independent variables possibly affecting ANS activity [7, 29, 30]. After adjusting for these confounders, we examined the relationship between all HRV components and drug formulation. The regression coefficients and their 95% confidence intervals were also calculated. Because the data were skewed, the absolute values of the HRV spectral components were log-transformed before statistical analysis.

All statistical analyses were performed using SPSS for Windows version 24 (IBM Corporation, Armonk, NY), with significance established at *p* < 0.05.

## Results

### Demographic and medication data of the participants

Table 1 shows demographic and medication data of the 72 patients receiving oral aripiprazole and the 50 patients receiving AOM (Table 1). There were no significant differences between the two groups in age, sex, duration of illness, BMI, and there were trend-level differences in aripiprazole dose, anticholinergic drug dose, and benzodiazepine dose (Table 1). There were no significant differences between the two groups in PANSS positive score (oral aripiprazole: 14.40 ± 3.51; AOM: 13.66 ± 3.58), PANSS negative score (oral aripiprazole: 19.49 ± 6.21; oral aripiprazole: 17:98 ± 6.07), or PANSS general psychopathology score (oral aripiprazole: 35.04 ± 9.68; AOM: 32.98 ± 9.50).

**Table 1:** Demographic and medication data of participants according to aripiprazole formulation prescribed (N = 122)

	Oral aripiprazole (n = 72)	AOM (n = 50)	*p* value
Age (years)	44.25 ± 13.56	41.72 ± 13.42	0.311
Sex (male/female)	30/42	21/29	0.971
Inpatient/outpatient	8/64	4/46	0.570
Duration of illness (years)	15.62 ± 10.58	12.50 ± 12.03	0.133
Smoking (smoker/non-smoker)	15/57	7/43	0.334
BMI (kg/m^2^)	24.81 ± 5.23	24.58 ± 5.63	0.814
CPZeq^a^ (mg/day)	392.01 ± 203.57	342.00 ± 89.19	0.068
BPDeq^b^ (mg/day)	0.82 ± 1.55	0.28 ± 1.47	0.054
DZPeq^c^ (mg/day)	6.16 ± 9.37	3.19 ± 8.84	0.081
PANSS total score	69.14 ± 16.94	64.78 ± 17.99	0.176

Data are presented as means ± standard deviations.

AOM, aripiprazole once-monthly; BMI, body mass index; PANSS, Positive and Negative Syndrome Scale; LAI, long-acting injectable.

Age, duration of illness, BMI, CPZeq, BPDeq, DZPeq, and PANSS total score were compared between the two groups using Student’s t-test.

Male/female, inpatient/outpatient, smoker/non-smoker proportions were compared using the chi-square test.

The proportions of men/women, inpatients/outpatients, and smokers/non-smokers were compared among the four groups using the chi-square test.

^a^ Daily dose of aripiprazole converted to an approximate chlorpromazine equivalent.

^b^ Daily dose of anticholinergic antiparkinsonian drugs converted to an approximate biperiden equivalent.

^c^ Daily dose of benzodiazepine converted to an approximate diazepam equivalent.

### ANS activities between the oral aripiprazole and AOM groups

Table 2 shows no significant differences in the TP or HF components between the two groups, but the LF component was significantly lower in patients receiving oral aripiprazole (*p* = 0.033) (Table 2).

**Table 2:** Comparison of log-transformed power values of the LF, HF, and TP frequency bands between patients who received oral aripiprazole and those who received AOM.

	Oral aripiprazole (n = 72)	AOM (n = 50)	*p* value
lnLF (ms^2^)	4.70 ± 1.22	5.21 ± 1.37	0.033 ^a^
lnHF (ms^2^)	4.27 ± 1.51	4.39 ± 1.49	0.672
lnTP (ms^2^)	5.32 ± 1.27	5.67 ± 1.33	0.152

Data are presented as means ± standard deviations.

AOM, aripiprazole once-monthly; ln, natural log-transformed; HF, high-frequency; LF, low-frequency; TP, total power; LAI, long-acting injectable.

^a^ Significant difference (*p* < 0.05; Student’s t-test).

### Association between ANS activities and clinical factors, including aripiprazole formulation

Table 3 shows the results of multiple regression analysis. Aripiprazole formulation was significantly associated with the LF component (*p* = 0.036), but not with the HF component or TP (Table 3). All of the HRV components were significantly associated with age. There were no associations of the spectral components of HRV with the other demographic and clinical factors.

**Table 3:** Multiple regression analysis using ANS activity, age, sex, body mass index, CPZeq, BPDeq, DZPeq, and aripiprazole formulation as independent variables

Independent variable	ANS activity								
	lnLF			lnHF			lnTP		
	β	95%CI	*p*	β	95%CI	*p*	β	95%CI	*p*
Age (year)	-0.364	-0.052 to -0.018	< 0.001^a^	-0.256	-0.050 to -0.008	0.007^a^	-0.343	-0.050 to -0.016	< 0.001^a^
Sex (reference category: female)	-0.038	-0.559 to 0.359	0.667	-0.005	-0.580 to 0.550	0.959	-0.023	-0.528 to 0.407	0.798
BMI (kg/m^2^)	-0.068	-0.059 to 0.026	0.444	-0.066	-0.071 to 0.033	0.477	-0.070	-0.060 to 0.026	0.435
CPZeq^b^ (mg/day)	-0.049	-0.001 to 0.002	0.541	-0.074	-0.002 to 0.001	0.433	-0.004	-0.001 to 0.001	0.965
BPDeq^c^ (mg/day)	0.071	-0.102 to 0.222	0.464	-0.064	-0.262 to 0.135	0.528	0.023	-0.145 to 0.184	0.816
DZPeq^d^ (mg/day)	0.058	-0.019 to 0.035	0.551	-0.010	-0.035 to 0.032	0.924	0.034	-0.023 to 0.033	0.731
Aripiprazole formulation (reference category: AOM)	-0.187	-0.952 to -0.034	0.036 ^a^	0.010	-0.535 to 0.594	0.917	-0.106	-0.746 to 0.189	0.240

ANS, autonomic nervous system; AOM, aripiprazole once-monthly; BMI, body mass index; ln, natural log-transformed; HF, high-frequency; LF, low-frequency; PANSS, Positive and Negative Syndrome Scale; TP, total power; CI, confidence intervals.

^a^ Significant difference (*p* < 0.05; multiple regression analysis).

^b^ Daily dose of aripiprazole converted to an approximate chlorpromazine equivalent.

^c^ Daily dose of anticholinergic antiparkinsonian drugs converted to an approximate biperiden equivalent.

^d^ Daily dose of benzodiazepines converted to an approximate diazepam equivalent.

## Discussion

This study analyzed the differential effects of oral aripiprazole monotherapy and AOM monotherapy on ANS activity in patients with schizophrenia. Significant differences in HRV were evident between patients receiving oral aripiprazole and those receiving AOM, with lower LF component values seen with oral aripiprazole. These findings suggest that patients taking oral aripiprazole have diminished sympathetic nervous activity, given that the LF component indicates sympathetic and partially vagal modulation. In other words, we found that AOM was associated with less sympathetic nervous system dysfunction compared with oral aripiprazole. Indeed, in our previous small-scale preliminary study, LF component values were lower in patients receiving oral antipsychotics than in those receiving LAIs [23]. Here, with a larger sample size, we obtained the same results in patients treated with aripiprazole monotherapy.

In recent studies, treatment-emergent adverse events resulting in the discontinuation of treatment were found to be similar in type and incidence between AOM and oral aripiprazole, and the safety and tolerability data suggested that both were well tolerated [31, 32]. However, as pointed out by Xiao et al. [31], several studies of psychotropic drugs have demonstrated that the risk of extrapyramidal symptom-related adverse events was lower with LAI antipsychotics than with tablets, due to the stable blood concentrations of drugs with long-acting formulations. The concentration of oral aripiprazole tends to be higher compared with the LAI equivalent [33], and the steady-state peak-to-trough plasma concentration ratios of antipsychotics vary according to drug type, with LAI antipsychotics having lower peak-to-trough blood level variations compared with oral antipsychotics [34, 35]. In addition, the physiochemical properties of aripiprazole lauroxil, which is a LAI prodrug of aripiprazole, result in slow dissolution and an extended pharmacokinetic profile, which affords flexibility in dose and dosing interval [36]. A significant association has been reported and investigated between plasma concentration and an increased risk of adverse events [37–39]. Moreover, a reduction in the peak concentration can mitigate the risk of dose-dependent side effects, while an increase in the trough concentration can mitigate the incidence of lack of efficacy due to subtherapeutic drug concentration [40]. We have also reported that antipsychotic drugs affect ANS activity in a dose-dependent manner [8]. Taken together, these factors could explain the advantage of AOM over oral aripiprazole in terms of adverse effects, such as sympathetic nervous system dysfunction.

The estimated bioavailability of AOM has been reported to be almost 48% higher than that of oral aripiprazole [41]. LAI antipsychotics ensure better bioavailability and a more predictable correlation between drug dose and plasma concentrations, allowing lower doses to be prescribed and reducing the risk of side effects [42]. In fact, in previous studies, the incidence of akathisia [31] and neuroleptic malignant syndrome [43] tended to be lower in patients receiving AOM than in those receiving oral aripiprazole. Because no previous studies had compared ANS activity between AOM and oral aripiprazole, we focused on determining whether AOM was associated with less sympathetic nervous system dysfunction than oral aripiprazole in the present study.

Notably, the advantage of AOM over oral aripiprazole in terms of treatment continuation was recently reported in the real-world clinical setting in Japan [44]. Moreover, patients with schizophrenia who were receiving LAIs antipsychotics were found to have a lower risk of death than patients receiving oral antipsychotics [45] and, in one analysis, the lowest incidence of cardiovascular deaths (0.17 per 1000 patient-years) was seen with atypical LAI antipsychotics [15]. We previously reported that decreased ANS activity might be associated with all-cause mortality in patients with schizophrenia [27], so our current finding that AOM was not associated with diminished ANS activity compared with oral aripiprazole might be associated with improved clinical safety as well as improved mortality. We did not investigate the effects of the aripiprazole formulation on treatment discontinuation or the incidence of cardiovascular death in this study and would need to monitor patients over the long term to examine those outcomes.

In multiple regression analysis, a significant association was seen between the aripiprazole formulation and the LF component related to ANS activity. The same association was found for age. However, age was unlikely to have influenced our finding that the aripiprazole formulation had different effects on sympathetic nervous system activity because there were no significant differences in age between the groups (Table 1).

This study has some limitations. First, the cross-sectional design means that causal relationships cannot be determined. Second, ANS activity might have varied due to various factors (e.g., treatment history) before the patients were assigned to the two study groups. Third, the group receiving AOM was small because we did not control for size differences in the patient groups. Fourth, trend-level differences in aripiprazole dose, anticholinergic dose, and benzodiazepine dose between the two groups might have affected the results, although multiple regression analysis showed that the drug formulation was associated with the LF component of HRV after adjusting for other clinical factors. Further studies are needed as confounding factors or small sample size might have affected the results of this study. Fifth, we evaluated drug adherence in oral aripiprazole by means of medical interviews alone. Adherence in the oral aripiprazole group might have affected the comparison between oral aripiprazole and AOM. Sixth, comparison between healthy controls and AOM was not investigated in this study. Finally, we were unable to investigate the serum and brain concentrations of aripiprazole in this study, and thus we did not evaluate the effect of changes in concentrations according to the medication time and dosing frequency of the oral drugs.

## Conclusion

In conclusion, AOM was associated with fewer side effects such as sympathetic nervous system dysfunction compared with oral aripiprazole. We need to monitor the course of patients with schizophrenia with diminished ANS activity who are taking aripiprazole, as well as compare the incidence of cardiovascular events more broadly, including cardiovascular deaths, between oral aripiprazole users and AOM users in a larger population. Studies of larger populations are also needed to further clarify the safety of AOM in relation to ANS activity in schizophrenia.

## Data Availability

The datasets used and analyzed in this study are available from the corresponding author on reasonable request.

## Abbreviations

ANS: autonomic nervous system
AOM: aripiprazole once-monthly,
BMI: body mass index
BPDeq: biperiden equivalent
CPZeq: chlorpromazine equivalent
DZPeq: diazepam equivalent
ECG: electrocardiography
EPS: extrapyramidal symptoms
HF: high-frequency
HRV: heart rate variability
LAI: long-acting injectable
LF: low-frequency
ln: natural log-transformed
PANSS: Positive and Negative Syndrome Scale
TP: Total power
